# The discovery of novel LPMO families with a new Hidden Markov model

**DOI:** 10.1186/s13104-017-2429-8

**Published:** 2017-02-21

**Authors:** Gerben P. Voshol, Erik Vijgenboom, Peter J. Punt

**Affiliations:** 1 Molecular Microbiology and Health, Institute of Biology Leiden, Leiden University, Leiden, The Netherlands; 2 Dutch DNA Biotech B.V., Utrechtseweg 48, 3703HE Zeist, The Netherlands

**Keywords:** Lytic polysaccharide mono-oxygenases, LPMO, Hidden Markov model, HMM, Fungi, Genome mining, Pectin, β-Glucan

## Abstract

**Background:**

Renewable biopolymers, such as cellulose, starch and chitin are highly resistance to enzymatic degradation. Therefore, there is a need to upgrade current degradation processes by including novel enzymes. Lytic polysaccharide mono-oxygenases (LPMOs) can disrupt recalcitrant biopolymers, thereby enhancing hydrolysis by conventional enzymes. However, novel LPMO families are difficult to identify using existing methods. Therefore, we developed a novel profile Hidden Markov model (HMM) and used it to mine genomes of ascomycetous fungi for novel LPMOs.

**Results:**

We constructed a structural alignment and verified that the alignment was correct. In the alignment we identified several known conserved features, such as the histidine brace and the N/Q/E-X-F/Y motif and previously unidentified conserved proline and glycine residues. These residues are distal from the active site, suggesting a role in structure rather than activity. The multiple protein alignment was subsequently used to build a profile Hidden Markov model. This model was initially tested on manually curated datasets and proved to be both sensitive (no false negatives) and specific (no false positives). In some of the genomes analyzed we identified a yet unknown LPMO family. This new family is mostly confined to the phyla of Ascomycota and Basidiomycota and the class of Oomycota. Genomic clustering indicated that at least some members might be involved in the degradation of β-glucans, while transcriptomic data suggested that others are possibly involved in the degradation of pectin.

**Conclusions:**

The newly developed profile hidden Markov Model was successfully used to mine fungal genomes for a novel family of LPMOs. However, the model is not limited to bacterial and fungal genomes. This is illustrated by the fact that the model was also able to identify another new LPMO family in *Drosophila melanogaster.* Furthermore, the Hidden Markov model was used to verify the more distant blast hits from the new fungal family of LPMOs, which belong to the Bivalves, Stony corals and Sea anemones. So this Hidden Markov model (Additional file 3) will help the broader scientific community in identifying other yet unknown LPMOs.

**Electronic supplementary material:**

The online version of this article (doi:10.1186/s13104-017-2429-8) contains supplementary material, which is available to authorized users.

## Background

Industrial biotechnology, also referred to as white biotechnology, is the application of living systems, or parts thereof (e.g. enzymes) for the environmentally friendly production and/or processing of industrially useful products [1]. For this purpose, industrial biotechnology uses widespread renewable biopolymers, such as cellulose, starch and chitin as feedstock. However, the resistance of these biopolymers to enzymatic, chemical and mechanical degradation limits their cost effective industrial use [2]. Therefore, there is a need to upgrade current degradation processes that are based on chemical or mechanical pretreatment followed by enzymatic hydrolysis by including novel enzymes.

Several approaches have been followed to attain improved enzyme cocktails. In our laboratory, as well as in many others, improved cocktails have been designed by combining known hydrolytic activities from different organisms such as fungi and cellulolytic Clostridia [3] and Streptomycetes [4]. In the 1970s it was already published that besides hydrolytic enzymes, oxygen requiring enzymes play an important role in the degradation of recalcitrant cellulose [5]. However, it wasn’t until 2010 that it was shown that metal dependent oxygenase enzymes, now known as lytic polysaccharide mono-oxygenases (LPMOs) are able to disrupt the structure of recalcitrant biopolymers, thereby opening it up for hydrolysis by conventional glycoside hydrolases [6, 7]. These LPMOs have since been reclassified in the carbohydrate-active enzymes database (http://www.cazy.org) [8] as the auxiliary activity family 9 (AA9; formerly GH61) and AA10 (formerly CBM33).

Since the initial discovery of the AA9 and AA10 families of LPMOs, Hemsworth and colleagues [9] used a “module walking” technique to discover a new family of LPMOs in 2013, the AA11s. Simply put, they used the modules attached to known LPMOs and searched for proteins which (i) contained that module, (ii) share only limited sequence homology to the known AA9 and AA10 families and (iii) contained a conserved histidine immediately after the signal peptide. A similar technique was later used by Vu and colleagues [10], who searched the genome of *Neurospora crassa* for (i) the conserved histidine after the signal peptide, (ii) a second conserved histidine and (iii) the N/Q/E-X-F/Y motif. Using this method, they identified 21 proteins, most belonging to the AA9 and AA11 families and one unknown which contained a CBM20 starch binding domain. This protein was subsequently shown to be a novel LPMO active against starch (the AA13 family [10, 11]).

In general, it is relatively easy to locate proteins putatively belonging to known LPMO families based on sequence homology, but as shown above it is much more difficult to accurately identify potentially novel LPMO families [11]. For example, a hidden Markov model based on an alignment of members of the AA13 family was only able to identify members of the AA13 family and not LPMOs belonging to the AA9, AA10 or AA11 family. Based on the increasing number of structural and biochemical characterizations of a growing number of LPMOs several conserved features were identified. First of all, all currently identified LPMOs share a similar core structure dominated by β-sandwich folds and a flat substrate binding surface [12, 13]. Secondly, until now, the histidine brace (1st and 2nd conserved histidine) that binds the copper using three nitrogen ligands is fully conserved in all members of all LPMO families [14–17].

In this study we developed a novel profile Hidden Markov model based on the structure of known LPMOs from the different families (AA9, AA10, AA11 and AA13) and used it to mine genomes of both actinomycetous bacteria (to verify the correctness of the model) and ascomycetous fungi for their full content of LPMOs.

## Methods

### Construction of the Hidden Markov model

A multiple protein alignment was created using the sequences indicated in Table 1 and the PROMALS3D structural alignment program (available at http://prodata.swmed.edu/promals3d/promals3d.php) without changing the default parameters [18]. The resulting multiple sequence alignment in clustal format was converted to stockholm format and used to build the Hidden Markov model using the esl-reformat and hmmbuild programs, respectively, which are part of the HMMER tool version 3 [19]. From the multiple protein alignment, a protein sequence logo was created using the java program LogoBar version 0.912 [20]. The frequency of the residues was represented by the size of the letter. Amino acids are grouped and colored using the following (cinema) color scheme, in which the polar positive amino acids are colored blue, the polar negative amino acids, red, the polar neutral amino acids green, the non-polar aliphatic amino acids grey, the non-polar aromatic amino acids magenta, brown or yellow. Gaps are indicated by bars. For brevity, regions where no clear consensus was observed were replaced by long vertical bars.

**Table 1:** Overview of characterized AA9, AA10, AA11 and AA13 enzymes used to build the Hidden Markov model

Organism	Uniprot ID	PDB ID	Substrate	Cleavage site	Auxiliary activity family	References
*Neurospora crassa* OR74A	Q1K8B6	4EIR	Cellulose	C4	AA9 (formerly GH61)	[11–13]
*Neurospora crassa* OR74A	Q7SA19	4EIS	Cellulose	C1, C4	AA9 (formerly GH61)	[11, 12]
*Phanerochaete chrysosporium* K-3	H1AE14	4B5Q	Cellulose	C1	AA9 (formerly GH61)	[14, 15]
*Thermoascus aurantiacus*	G3XAP7	2YET	Cellulose	C1	AA9 (formerly GH61)	[16]
*Enterococcus faecalis* V583	Q838S1	4A02	Chitin	C1	AA10 (formerly CBM33)	[4, 17]
*Burkholderia pseudomallei* 1710b	Q3JY22	3UAM	n.d.	n.d.	AA10 (formerly CBM33)	[18]
*Vibrio cholerae* O1 biovar El Tor str. N16961	Q9KLD5	2XWX	n.d.	n.d.	AA10 (formerly CBM33)	[19]
*Bacillus amyloliquefaciens* DSM7	E1UUV3	2YOX	n.d.	n.d.	AA10 (formerly CBM33)	[20]
*Serratia marcescens* BJL200	O83009	2BEM	Chitin	C1	AA10 (formerly CBM33)	[4–6]
*Streptomyces coelicolor* A3(2)	Q9RJC1	4OY6	Cellulose, chitin	C1, C4	AA10 (formerly CBM33)	[7]
*Streptomyces coelicolor* A3(2)	Q9RJY2	4OY7	Cellulose	C1, C4	AA10 (formerly CBM33)	[6–8]
*Thermobifida fusca* YX	Q47QG3	4GBO	Cellulose, chitin	C1, C4	AA10 (formerly CBM33)	[7]
*Aspergillus oryzae* RIB40	Q2UA85	4MAI	chitin	C1	AA11	[9]
*Aspergillus oryzae* RIB40	Q2U8Y3	4OPB	n.d.	n.d.	AA13	[10]

The identified conserved residues were subsequently analyzed visually using the 3d molecular graphics program CCP4mg version 2.10.6 [21].

### Genome mining for LPMOs

Genomes indicated in Table 2 were downloaded from their respective databases (EMBL, Genbank or JGI) and searched using the newly build Hidden Markov model and the hmmsearch tool [19]. Before being able to determine which hmmsearch hits are potentially interesting to look at, one needs to determine a trusted cutoff e-value. This e-value can easily be determined by searching the sequences of the 45 LPMOs (using the hmmsearch program) that have been fully or partially characterized (available from http://www.cazy.org) and take the e-value of the lowest scoring true positive. This resulted in an e-value of 1.2e−15, everything that scores lower than this value are most probably LPMOs belonging to the AA9, AA10, AA11 or AA13 families and 1 in 1.2e15 is expected to be a false positive. Using this e-value 96% of the LPMOs identified in this study and verified using a combination of BLAST, HMM searches and other forms of manual curation (e.g. presence of histidine brace, signal sequence, conserved motif), were detected. To detect the remaining LPMOs, we looked at those hits, just below the trusted E-value (<1.2e−15) and manually verified those hits. For example, a novel LPMO from *Aspergillus niger (An01g12440)* has an e-value of 2.5e−15, which is just below the trusted e-value of 1.2e−15. After manual curation and including these novel LPMOs, the E-value could be changed to a new (default) trusted e-value of 0.001 (which theoretically results in a 1 in 1000 false positive discovery rate). Using this threshold, all known LPMOs could be identified (except 4 out of 1399 sequences in CAZY, which turned out to be incorrectly annotated) and new LMPO families which we subjected to further analysis in this paper. It should be noted that performing the Hidden Markov model search without pre-filtering the sequences is required to find all known members of the AA9 family. This is due to the bias composition filter, which checks sequences for biased residue composition (e.g. large regions of hydrophobicity, repetitive regions, etc.) and therefore tends to remove some members of the AA9 family. For example, with bias filtering 17 out of 19 LPMOs were identified in the genome of *N. crassa* OR74a because both NCU01867 and NCU07974 were removed by the bias filter.

**Table 2:** LPMO families of mined fungal genomes

Strain	LPMO family				
	AA9	AA11	AA13	LPMO14	Total
*Aspergilus niger* CBS 513.88	7	3	0	1	11
*Aspergillus oryzae* RIB40	8	5	1	2	16
*Aspergillus nidulans* FGSC A4	9	2	2	1	14
*Trichoderma reesei* v.2.0	3	3	0	0	6
*Neurospora crassa* OR74A	14	4	1	0	19
*Myceliophtora thermophile* ATCC 42464	22	4	1	3	30

### In silico functional analysis of new LPMO families

To elucidate the putative function of the new LPMO family members, two in vitro approaches were followed. (i) The clustering of the new LPMO family genes with genes of known function was examined using the precomputed Jaccard clusters from the *Aspergillus* Genome Database [22] and (ii) the transcriptional profile of the genes encoding the identified novel LPMO proteins of interest were analyzed. Transcriptomic data was obtained using the publically available data located in the ArrayExpress expression database [23] or other cited literature sources. The Babelomics tool version 4.3 (available at http://v4.babelomics.org/) was used to cluster genes together based on their transcription with the k-means clustering method (k-value = 100), using the Euclidean expression distance (normal) and the unweighted pair group method with arithmetic mean grouping [24].

## Results

### The Hidden Markov model

The quality of the generated Hidden Markov model is significantly influenced by the initial multiple protein alignment and therefore requires manual curation to verify that the alignment is correct [25]. This is especially true for alignments generated by using highly divergent sequences, such as the LPMO sequences used to build the model for the research described here, where alignment algorithms do not perform very well [26, 27].

**Fig. 1:**
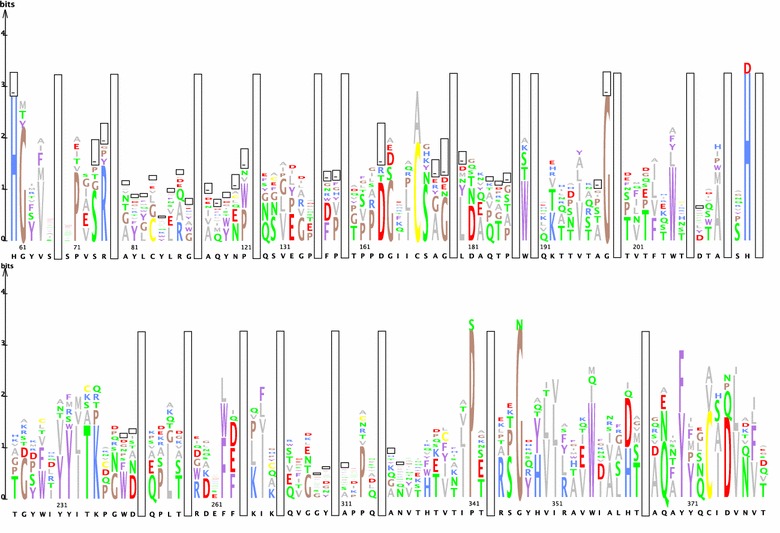
A protein sequence logo constructed from the multiple alignment of selected LPMOs. The frequency of the residues is represented by the size of the letter. Amino acids are grouped and colored using the cinema color scheme. Gaps are indicated by *bars*. For brevity, regions where no clear consensus was observed were replaced by *long vertical bars*

**Fig. 2:**
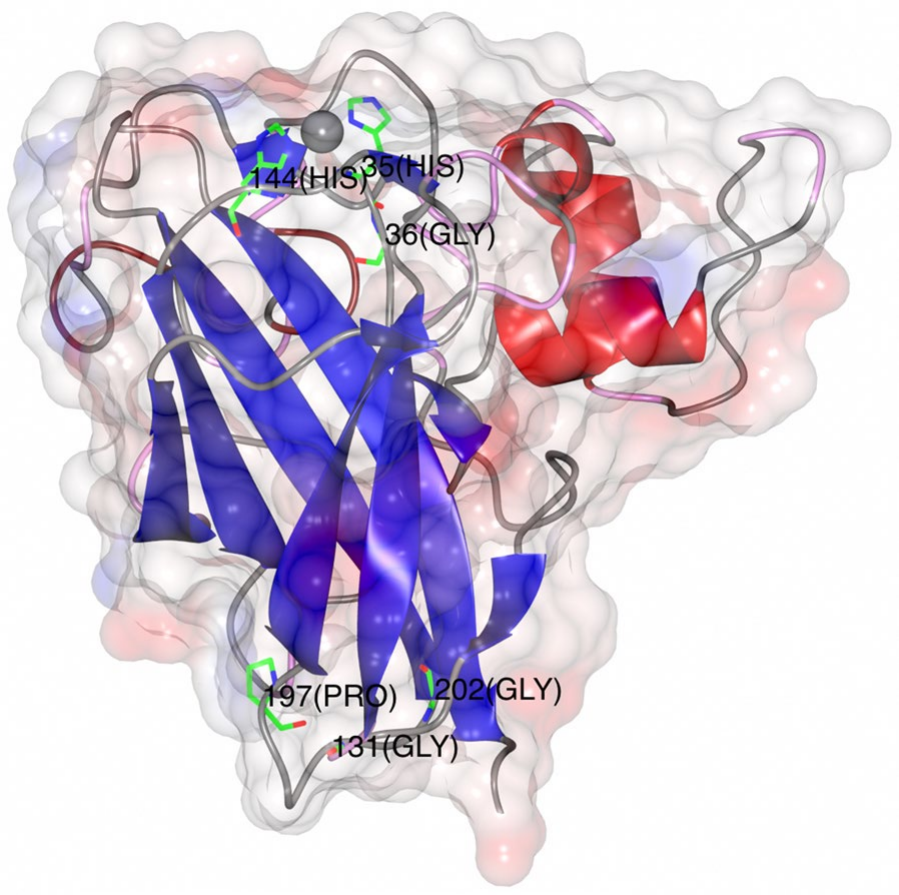
Three-dimensional structure of ScLPMO10C indicating several highly conserved residues. The structure of one of the AA10 LPMOs from *Streptomyces coelicolor* A3(2) (PDB ID: 4OY7) [39], with the copper atom shown as a *sphere* and highly conserved residues labeled and shown as *sticks*

To visually inspect the quality of the multiple sequence alignment we generated a protein sequence logo (see Fig. 1). In general, only a very small number of amino acids is conserved between the four families of LPMOs. Nevertheless, one of the most important features that needs to be present in any functional multiple protein alignment of LPMOs, the histidine brace [9, 17], can easily be seen in the sequence logo at location 60 and 224. Moreover, the conserved N/Q/E-X-F/Y motif (located between amino acid 386 and 370), earlier used by Vu and colleagues [10], is also present in the sequence logo. Nevertheless, in the constructed multiple protein alignment, the AA10 from *Vibrio cholerae* [28] has an alanine instead of the more common asparagine, glutamine or glutamic acid residue. Besides these features, there are also three highly conserved glycines and a single proline. Unexpectedly, almost all of these residues with the exception of the first glycine are located distal from the active site (see Fig. 2) suggesting a role in structure rather than in activity.

### Genome mining for LPMOs

Another important measure of quality control is the ability of the model to accurately identify known LPMOs without any false positives. Therefore, the new Hidden Markov model was first used to screen the genome of *Streptomyces lividans* 1326 [29], the parental strain of *S. lividans* TK24, which was already previously scrutinized for its LPMOome [30]. All known LPMOs were identified in the genome of *S. lividans* 1326 using our Hidden Markov model and no other sequences were detected. Subsequently, the Hidden Markov model was used for screening the genomes of several industrially important filamentous fungi (Table 2). This resulted in the identification of all previously identified fungal LPMO families AA9, AA11 and AA13. Surprisingly, we identified a completely new family of LPMOs, which was tentatively named LPMO14.

**Fig. 3:**
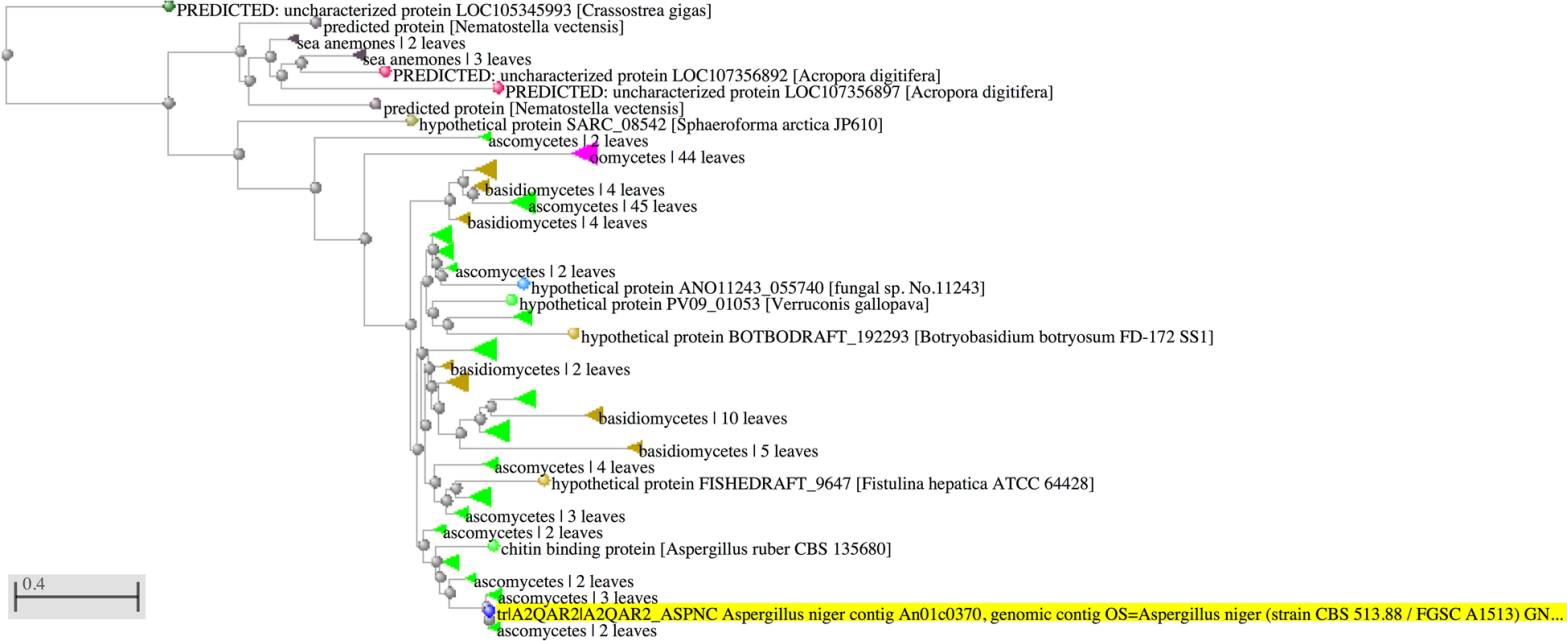
BLAST tree view showing the taxonomic distribution of the new LPMO14 family. The sequence (An01g12440; LPMO14) highlighted in *yellow* was used as the blast query. The tree was constructed using Fast Minimal Evolution with a maximum sequence difference of 0.9 and Grishin was used to calculate the protein distances. Branches are collapsed and colored as follows, Bivalves in *dark green*, Sea anemones in *grey*, Stony corals in *red*, Eukaryotes in *dark brown*, Ascomycetes in *light green*, Oomycetes in *purple*, Basidiomycetes in *light brown*, Fungi in *light blue* and unknown sequences in *dark blue*

Based on the identified new family members we also performed BLAST searches against all protein sequences available at NCBI. The resulting BLAST tree view revealed that this novel family has a limited taxonomic occurrence (Fig. 3). Approximately 84% of all the BLAST hits belonged to the kingdom of Fungi, followed by 14% which belonged to the kingdom of Protista and the final 2% belonged to the kingdom of Animalia. Most of the hits belonging to the fungal kingdom belonged to either the Phylum of Ascomycota (81%) or Basidiomycota (19%). Almost all of the hits (99%) from the kingdom of Protista belonged to the genus *Phytophthor*a, a plant pathogenic genus which occupies a very similar ecological niche as phytopathogenic Ascomycota. The other hits belonged to either the Bivalves, Sea anemones or Stony corals.

**Fig. 4:**
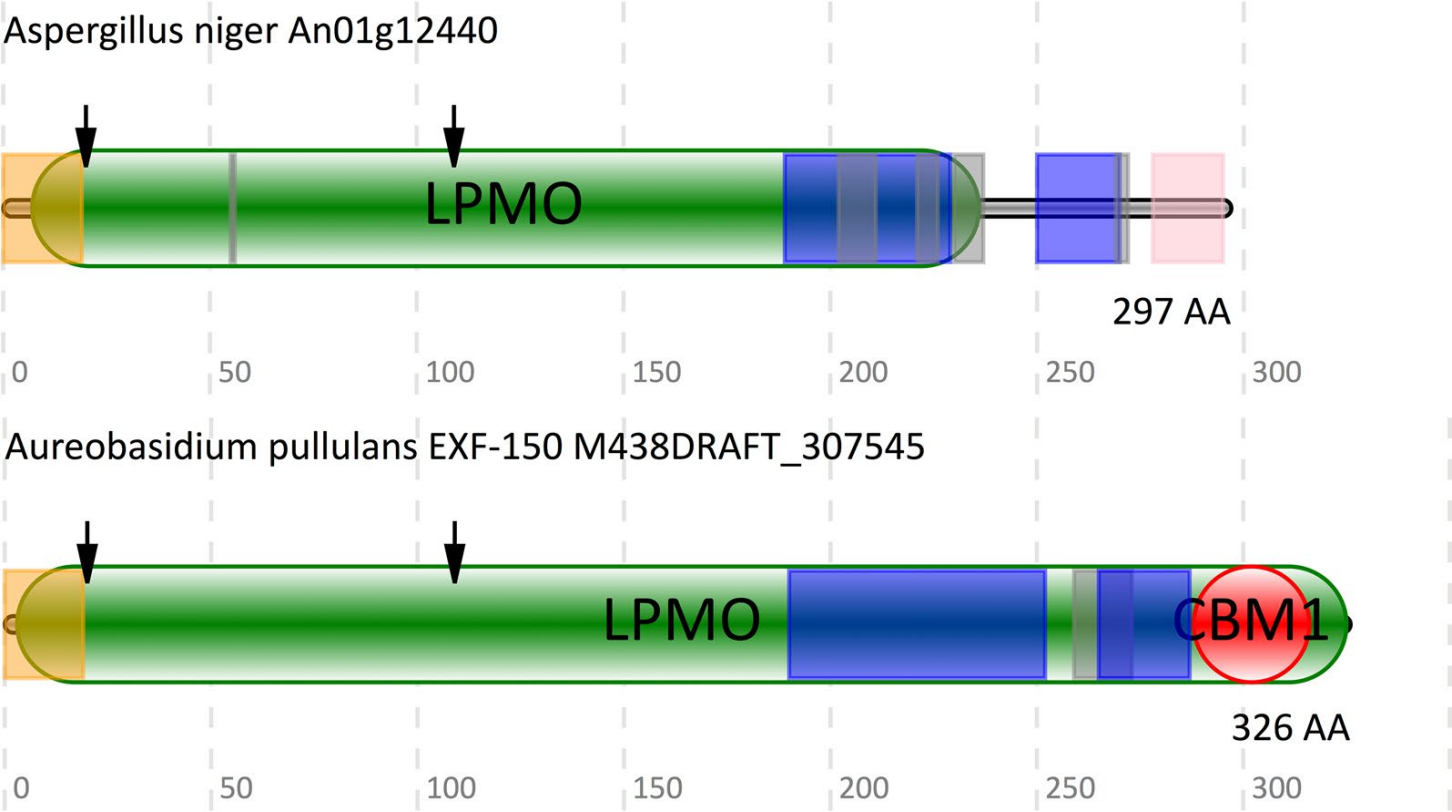
A schematic overview of the domain organization of representative LPMOs belonging to the new LPMO14 family. The signal peptide is indicated in *orange*, low complexity regions in *blue*, disordered regions in *grey*, the LPMO domain in *green*, the carbohydrate binding module 1 (CBM1) in *red* and the LPMO14 helix in *pink*. The conserved histidine residues are indicated with *arrows*

A more detailed analysis of all genes encoding LPMO14 family members revealed that a large number (562 out of 566) of them also encoded a secretion signal sequence (Fig. 4), followed by the conserved LPMO14 histidine brace, an extended S/T/A rich linker region and a conserved helical domain possibly representing a novel substrate binding domain. A few of the LPMO14 family members had a previously identified carbohydrate binding domain of the CBM1 family (Fig. 4). It should be noted that SignalP [31] was unable to unambiguously detect a secretion signal in two out of four of these proteins, but all other required features (e.g. the histidine brace) are present. Furthermore, it appears that the CBM1 domain overlaps with new Hidden Markov model (LPMO domain), outside of the core LPMO domain.

### Putative function of the new LPMO14 family

To elucidate the putative function of the LPMO14 family members, an in silico approach was followed by examining the possible grouping of LPMO14 encoding genes in gene clusters related to specific carbohydrate polymer degradation. Interestingly, in almost all *Aspergillus* species examined the LPMO14 encoding gene is within a Jaccard gene cluster containing a total of 19 genes. Inside this cluster directly adjacent to the LPMO14 gene there is another gene involved in carbohydrate degradation, namely orthologues of gene An01g12450. This gene encodes a protein containing two pectin lyase domains and belongs to the glycoside hydrolase family 55 [8]. This family contains members having exo-β-1,3-glucanase activity, which are involved in the degradation of β-1,3-glucans. β-1,3-glucans are polysaccharides typically found as chrysolaminarin, lichenin, callose, etc. The clustering of the LPMO14 family members with this gene suggests that this LPMO14 member might be involved in the degradation of β-glucan containing biopolymers [32].

Since the expression of the LPMO14 gene from *A. niger (An01g12440*) is low in the variety of expression data we examined [33] this precludes further indication on its function. To get another indication for LPMO14 function, we focused on the three orthologues from *Myceliophtora thermophile* ATCC 42464 for which expression data are available. One of the added advantages of this approach is that we can study three members of the same family at the same time. Out of the three LPMO14 members, protein MYCTH_103070 shares the highest percentage of sequence identity with the *A. niger* orthologue An01g12440 (54%), followed by MYCTH_2311254 (48%) and MYCTH_2306267 (36%). K-means clustering of the genes was performed using the transcriptomic data of Kolbusz and colleagues, which grew *M. thermophile* ATCC 42464 on six different complex carbon sources barley, oat, triticale, alfalfa, canola, flax and glucose [34, 35]. The results (see Additional file 1) indicated that gene MYCTH_103070 (the most similar orthologue of An01g12440) clusters together with MYCTH_66804 and MYCTH_58642, which encode a glycoside hydrolase family 3 and a sugar transport like protein, respectively. The glycoside hydrolase family 3 contains members with diverse activities including α-l-arabinofuranosidase, xylan 1,4-β-xylosidase and glucan 1,3-β-glucosidase. Another LPMO14 family member, MYCTH_2306267, clusters together with MYCTH_52713 and MYCTH_90594 both encoding polysaccharide lyase proteins involved in the degradation of pectin. The expression of the remaining LPMO14, MYCTH_2311254 is low, thus it does not cluster into a well-defined group.

## Discussion

In this study we describe the construction of a novel Hidden Markov model and its use in the mining of fungal genomes for LPMOs. The model was initially validated by the presence of several essential features, such as the histidine brace and the N/Q/E-X-F/Y motif in the active site. Besides the histidine brace, we also identified several conserved proline and glycine residues distal from the active site (Fig. 2). These newly identified residues were previously overlooked and are a 100% conserved in all examined LPMOs, indicating their importance. Some more variable residues, such as those involved in substrate binding, are less conserved between different LPMO families (Fig. 1). For example, the triple aromatic tyrosine residues of the AA9 family [36] are hardly visible in the protein sequence logo, while the tryptophan residue at location 355 has a high bit score. This difference in substrate binding residues between families can be exploited for the automated in silico determination of LPMO substrate specificity. Earlier, Busk and Lange used a similar approach to sort protein sequences into their respective LPMO families [37]. Although classification of putative LPMO proteins into different families is important, a finer grained grouping based on substrate specificities should further aid in identifying LPMOs with yet unknown activities.

After the initial validation of the model, we also confirmed its ability to accurately identify all known protein members contained by the different LPMO families. Our newly developed model is able to identify all LPMOs without any false positives or negatives (in the 8 genomes examined in this study). This is not true for other methods currently used to mine genomes for LPMOs. For example, using the LPMO_10 Pfam Hidden Markov model (PF03067), we were able to identify only 4 out of the 30 LPMOs from the genome of *M. thermophile* ATCC 42464. It should be noted that the Pfam model was constructed using only bacterial LPMO protein sequences and therefore it most likely led to the failure to identify 26 LPMOs. Vu and colleagues, using a different method, were able to identify all of the LPMO genes in *N. crassa* OR74A however, they also identified 3 false positives. Moreover, they relied on the ability of signalP to correctly identify the location of the signal peptide. However, as indicated above (Fig. 4), some of the members of the LPMO14 family have a very weak signal cleavage site (located at the first histidine), which would have been missed if searched for using such a method.

Although for this study we limited ourselves to searching a small selection of fungal genomes, the Hidden Markov model is not limited to bacterial and fungal genomes. This is illustrated by the fact that after searching the *Drosophila melanogaster* genome, another putative family of LPMOs was identified, represented by 3 genes (gene CG4367, CG4362 and CG42749). Furthermore, lowering the HMM threshold e-value from the default 0.001–0.01 (which increases sensitivity but also the theoretical incidence of false positives to 1 in a 100 queries) yet another group of putative LPMOs, represented by *A. niger* An07g08250, was discovered. Although the amino acid sequence similarity to the known LPMO families is very low, a structure based search (https://swissmodel.expasy.org) shows its closest structural orthologue to be a member of the AA9 family. Whilst a more thorough discussion of these putative new families is beyond the scope of this short research note, it implies the broad applicability and power of the developed Hidden Markov model. The results further indicate that LPMOs are ubiquitously present in all kingdoms of life, including Animalia, Bacteria, Fungi, Planta and Protista.

The substrate specificity of the identified LPMO14 family members was predicted using an in silico approach. Firstly, a genomic clustering approach was used to identify genes which occurred in the same Jaccard cluster. This clustering analysis indicated that An01g12440, clustered with a GH55 glycoside hydrolase (exo-β-1,3-glucanase) containing two pectin lyase domains. Secondly, the genomic clustering was supported by a detailed analysis of the transcriptomic data of *M. thermophile* ATCC 42464. A k-means clustering using this transcriptomic data, indicated that the most similar orthologue of An01g12440, namely MYCTH_103070, clustered with a member of the GH3 family (Additional file 1). The GH3 family, similarly to the GH55 family, includes enzymes with several activities including β-glucanases. These genomic and transcriptomic results correlate well with the observation that several members of the LPMO14 family contain a CBM1 domain (Fig. 4). The CBM1 family has been shown to bind cellulose [38], which is also a β-glucan. Taken together these results indicate that the most similar orthologues of An01g12440 are most likely involved in the degradation of glucan containing biopolymer.

K-means clustering of the transcriptomic data of *M. thermophile* ATCC 42464 led to another very interesting observation. Although the most similar orthologue of An01g12440 clustered with a glucanase, the more distant LPMO14 family member of *M. thermophile* ATCC 42464 (MYCTH_2306267) clustered with several pectinolytic enzymes. This result seems to suggest that the LPMO14 family may have members with several substrate specificities, namely glucan and pectin. Interestingly and in line with this observation, a tree constructed using the different LPMO family members identified in this study supports a separation of the LPMO14 family into at least two subfamilies (Additional file 2).

## Conclusion

In conclusion, this newly developed Hidden Markov model can be used for the mining of genomes, metagenomes and transcriptomes for known and novel LPMOs. Furthermore, we demonstrated that the model is not limited to a single phylogenetic group, but is able to correctly identify LPMOs in several distinct kingdoms (e.g. Bacteria, Fungi, Animalia, etc.). Moreover, using this approach, we were able to identify two families of LPMOs which were previously unidentified. We hope that this paper and its Hidden Markov model (Additional file 3) will help the broader scientific community in identifying other yet unknown LPMOs.

With respect to the newly identified LPMO14 family, our ongoing efforts are focused on expression and characterization studies of several of its family members. In future studies it could also be interesting to elucidate the function of the newly identified conserved proline and glycine residues.

## Additional files

**Additional file 1.** K-means clusters of two LPMO14 genes from *Myceliophtora thermophile* ATCC 42464 (MYCTH_103070 and MYCTH_2306267). K-means clustering was performed using the Babelomics tool (http://babelomics.org) with transcriptomic data of Kolbusz and colleagues, which grew *Myceliophtora thermophile* ATCC 42464 on six different complex carbon sources and glucose.

**Additional file 2.** Phylogenetic tree of all fungal LPMOs identified in this study. An alignment of fungal LPMO sequences identified in this paper was performed using Muscle (v3.8.31), clustering was performed using phyML and the final tree was rendered using the TreeDyn program.

**Additional file 3.** The Hidden Markov model constructed in this study. This Hidden Markov model, can be used in combination with the HMMER tool version 3 [19]. When searching with the hmmsearch program, using a trusted e-value (-E option in hmmsearch) of 1.2e−15 (lowest scoring true positive) will limit the results to mainly AA9, AA10, AA11 and AA13 LPMOs. To also allow the detection of the LPMO14 family, a lower trusted e-value of 0.001 is necessary. However, it should be noted that a lower e-value will also increase the theoretical chance of finding false positives (an e-value of 0.001, has a 1 in a 1000 chance to find a false positive). However, in our search using this e-value no false positives were identified in the 8 genomes searched.

**Additional file 4.** UniProt/PDB identifiers of the genes used in this study and links to the NCBI Genomes FTP site containing the protein sequences of the genomes listed in this study.

## Abbreviations

LPMO: lytic polysaccharide mono-oxygenase
AA: auxiliary activity family
CBM: carbohydrate binding module
HMM: Hidden Markov model
