# Supplementary figures and images for: The discovery of novel LPMO families with a new Hidden Markov model
Source: BMC Res Notes. 2017 Feb 21;10:105. doi: 10.1186/s13104-017-2429-8 (PMC5320794; doi:10.1186/s13104-017-2429-8)

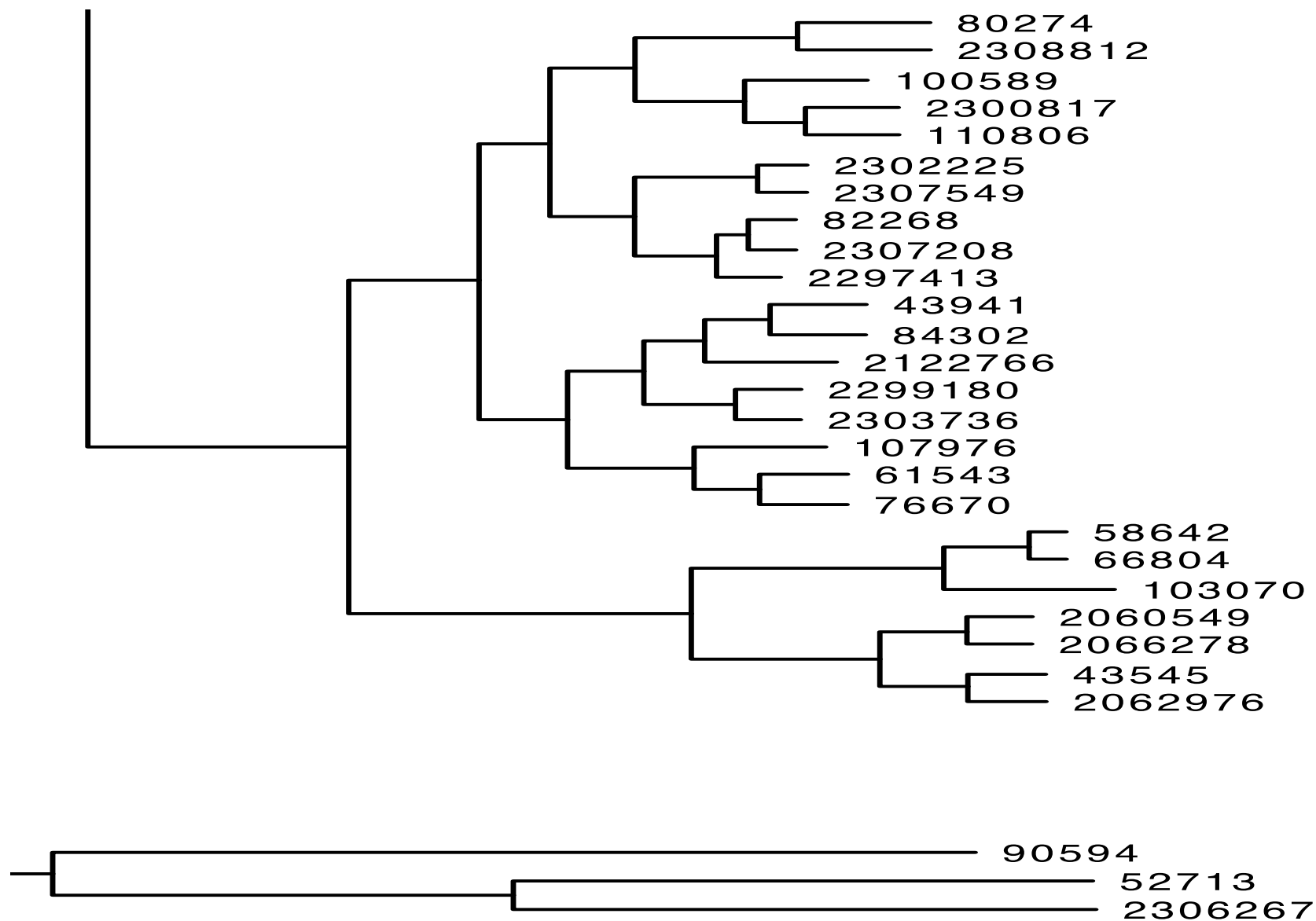

Supplement: Supplementary file 1 — Additional file 1. K-means clusters of two LPMO14 genes from Myceliophtora thermophile ATCC 42464 (MYCTH_103070 and MYCTH_2306267). K-means clustering was performed using the Babelomics tool (http://babelomics.org) with transcriptomic data of Kolbusz and colleagues, which grew Myceliophtora thermophile ATCC 42464 on six different complex carbon sources and glucose. [file 13104_2017_2429_MOESM1_ESM.pdf]

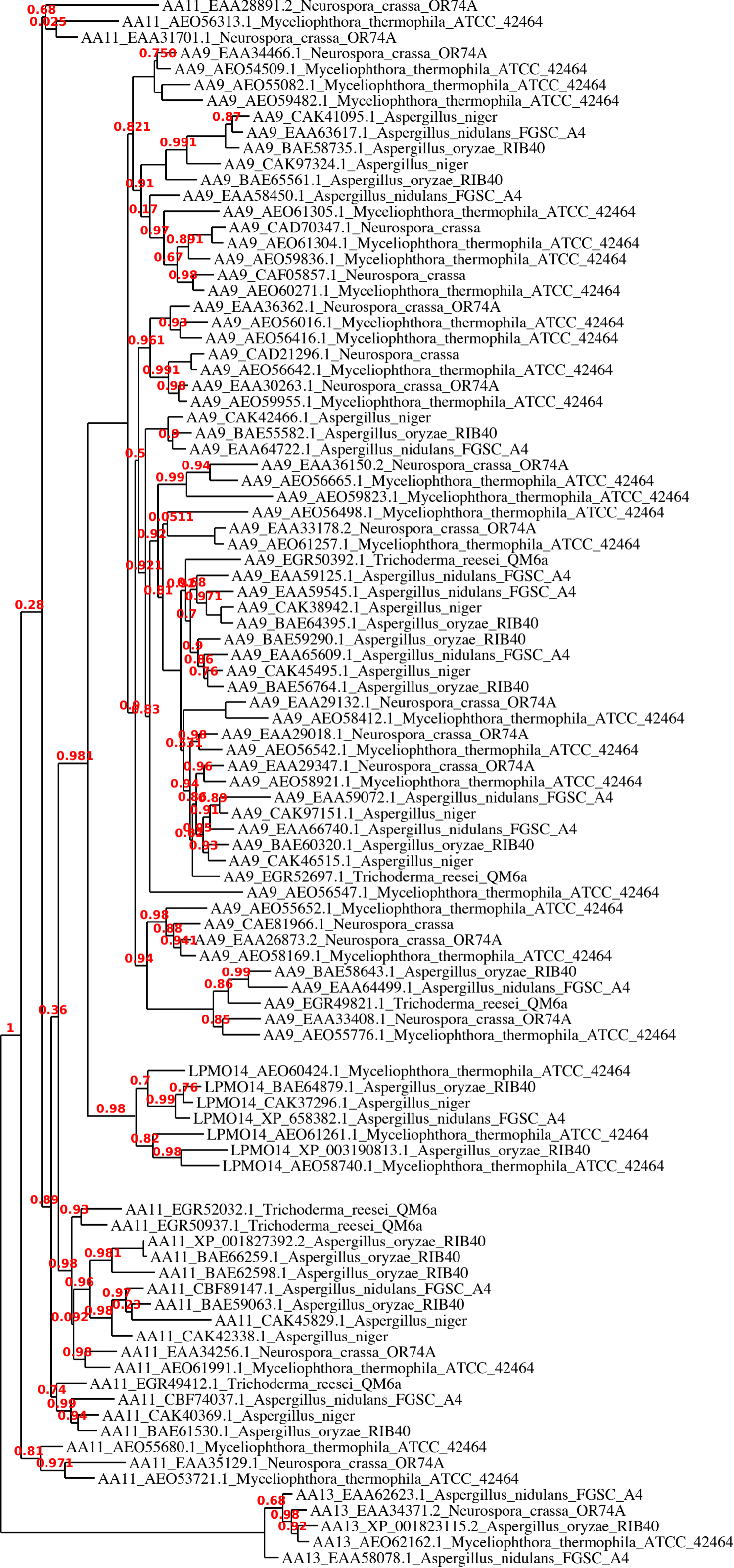

Supplement: Supplementary file 2 — Additional file 2. Phylogenetic tree of all fungal LPMOs identified in this study. An alignment of fungal LPMO sequences identified in this paper was performed using Muscle (v3.8.31), clustering was performed using phyML and the final tree was rendered using the TreeDyn program. [file 13104_2017_2429_MOESM2_ESM.pdf]
